# Determinant factors of Chief Data Officer adoption in government: A topic model and structural equation modelling approach

**DOI:** 10.1371/journal.pone.0328683

**Published:** 2025-08-28

**Authors:** Hui Zhang, Huiying Ding, Jianying Xiao

**Affiliations:** School of Public Policy & Management, China University of Mining and Technology, Xuzhou, Jiangsu, People's Republic of China; Purdue University, UNITED STATES OF AMERICA

## Abstract

With the generation of massive amounts of data, the Chief Data Officer (CDO) has been introduced in governments worldwide. Existing research on CDO is quite limited and primarily focuses on general descriptions of CDO. However, there is little research exploring the underlying reasons for the establishment of the CDO in government. To address this gap, this paper employs topic modeling to analyze government documents, identify factors influencing the adoption of CDO, and construct a research model. Data were collected from 277 employees within Chinese government organizations through a questionnaire survey and a quantitative analysis was performed to evaluate five hypotheses using structural equation modeling (SEM). The findings suggest that (1) data exploitation, data sharing and data management significantly influence data dividends, and (2) both data dividends and institutional pressures are key predictors of the intention to adopt the CDO, with digital dividends exerting a greater effect than institutional pressures.

## Introduction

The application of digital techniques and the digitization of governments and societies have resulted in the generation of vast amounts of data [1]. Data has emerged as a powerful strategic resource for government organizations [2]. Consequently, governments require dedicated personnel to manage data [3]. The CDO has been introduced in governments around the world to unlock the value of data [3–5]. The main responsibility of a CDO is to develop and implement data-related policies and governance [5,7]. Thus, the establishment of the CDO can enhance the development of the digital economy [4], improve quality of public services and government service efficiency [5], and bolster data security [6].

The realization of these benefits hinges on the adoption of the CDO by government organizations. Existing literature on the CDO within the government is quite limited, primarily focusing on the responsibilities [5,7] and the relationship between the CDO and Chief Information Officer (CIO) [5,7,8]. However, there has been insufficient research addressing the underlying reasons for establishing of the CDO. Understanding the adoption of systems or initiatives within government organizations is crucial for their success [9,10] and serves as a predictor of organization’s future behavior [11]. This principle is equally applicable to the CDO initiative. Furthermore, there is a scarcity of empirical research concerning the CDO.

To address this gap, the primary research questions of this study are: (1) What factors influence the adoption of the CDO in government organizations? (2) What prominent factors determine the intention to adopt? To explore these questions, a topic model and SEM are employed to propose a model outlining the factors that impact the adoption of the CDO.

This study makes three significant contributions to the research on CDO. First, it expands the existing literature on CDO by providing a parsimonious model that elucidates how five factors influence the adoption of CDO in government. Second, the study offers a detailed exposition of topic model, specifically applied to CDO-related literature. Third, it develops four-item scales to measure data dividends, addressing a gap in the literature, as there is currently only a conceptual description of data dividends without an established measurement scale.

## Literature review

To extract the value of data and achieve a data-driven government, the primary responsibility of the CDO is to develop and implement data-related policies and governance [5,7,12]. There are differences and connections between the CDO and CIO. First, the CDO and CIO emerged from distinct technological revolutions: the computing revolution and the data revolution, respectively [8]. Their establishment aims to address the challenges and opportunities presented by technology at different historical junctures [13]. Second, their responsibilities differ significantly: the CDO serves as the data leader, concentrating on data policy and governance [5,7], whereas the CIO functions as the IT leader, focusing on IT policy and governance [14]. Third, the CDO must collaborate with the CIO and other senior managers on data policy [5], digital strategies and roadmaps [4], and execution of digital transformation initiatives [15]. Their establishment can significantly enhance organizational performance [13,15].

Previous studies have laid the foundation for subsequent research; however, they also exhibit certain limitations. First, while prior studies primarily provided general descriptions of CDO, little research has explored the factors influencing the adoption of the CDO. Second, earlier studies predominantly employed qualitative methods, resulting in a scarcity of empirical research concerning the CDO in government.

### Research models and hypothesis

#### Variable source.

To identify the factors influencing the appointment of the CDO within the context of CDO-related documents, this study employs a topic model approach. Specifically, the study utilizes the Latent Dirichlet Allocation (LDA) algorithm to extract topics and determine the optimal number of topics.

#### Topic model.

With the application of digital technology in academic research, some researchers have introduced statistical algorithms for the analysis of text documents [16,17], leading to the development of new methods known as topic models. Topic model is defined as “a suite of algorithms whose aim is to discover the hidden thematic structure in large archives of documents” [16]. Thus, topic model can identify potential topics within a given text collection [18] and effectively handle unclassified textual data sources and unstructured digital text [19]. Topic model possess two significant characteristics: (1) they utilize machine learning techniques to impose structure on the text [20], and (2) they encode the content of text by an automated procedure [21].

The topic model has been introduced into the field of public administration due to its advantages [22,23]. Despite the growing interest among scholars in public administration regarding topic model, research utilizing this approach remains limited. To broaden the application scope of topic model, this article applies it to the issue of the CDO.

**Fig 1:**
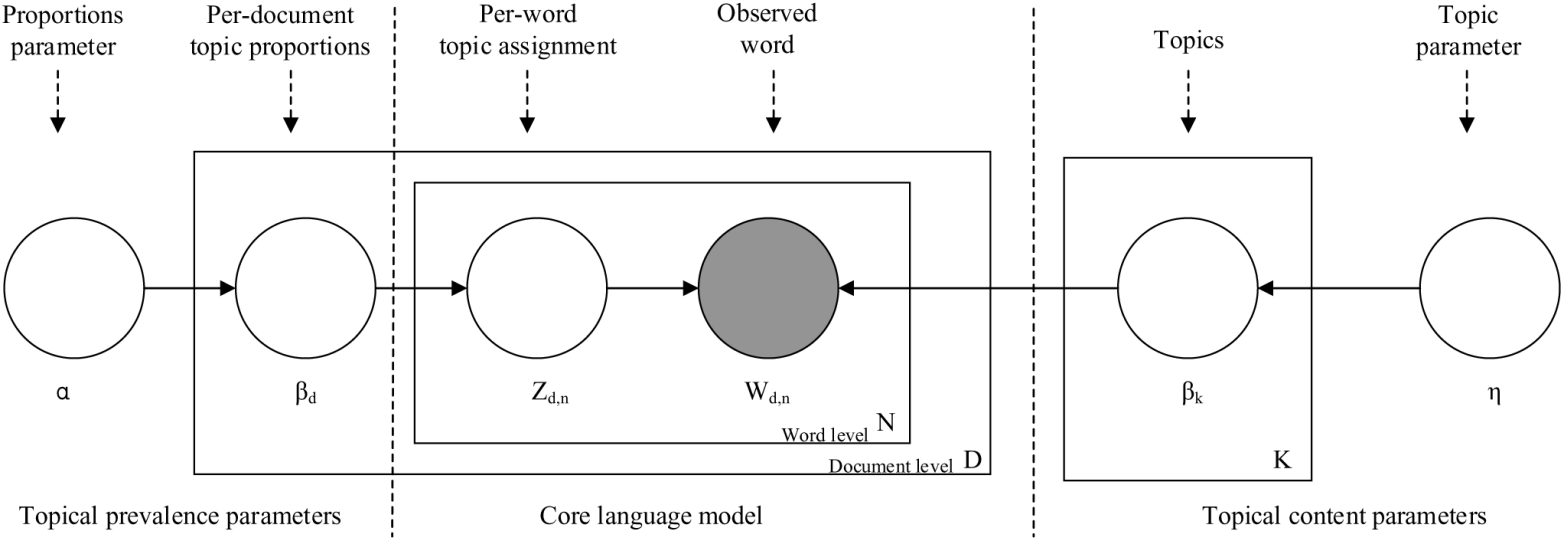
LDA algorithm [11,28,31,59].

LDA is one of the simplest generative models used for topic model [24]. It conceptualizes documents as multinomial distributions over latent topics, with each topic is characterized by a distribution over words [24,25]. The process of LDA is illustrated in Fig 1.

In this study, LDA was employed to analyze the collected policy texts, and the identified topics were considered as factors influencing CDO adoption. The specific steps of the analysis are as follows:

### Documents collection

**Fig 2:**
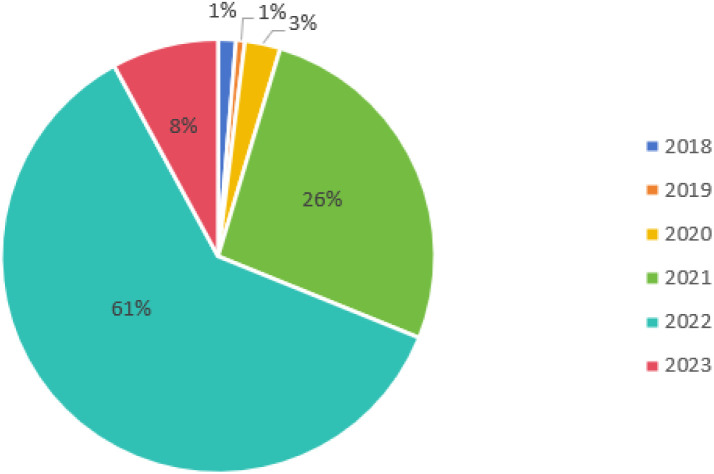
Government documents about CDO in 2018-2023.

Selecting a corpus is essential, as LDA is an inductive methodology [22,23,26]. Descriptions of the CDO can be found in government documents pertaining to the CDO system, data governance, the marketization configuration of data elements, public data resource management, open government data, big data, digital government, and government digital transformation, among others. This study constructed a consisting corpus of 604 government documents related to the CDO, covering the period from January 2018 to March 2023. The data preprocessing involved two steps: First, given that the focus of this study is on the CDO, documents that did not mention the CDO were removed (n = 334). Second, since this study centers on the factors influencing CDO appointments, documents that merely referenced the CDO were also excluded (n = 119). Ultimately, 151 documents met the criteria for inclusion in this study. The year and number of government documents about the CDO are presented in Fig 2.

As shown in Fig 2, government documents concerning CDO first emerged in 2018. Since 2019, the quantity of such documents has consistently increased annually, peaking in 2022 before experiencing a decline in 2023.

Prior to formal analysis, this study compiled terms specific to the policy documents into a personal dictionary, which was subsequently added to the jieba word segmentation list. High-frequency but semantically void terms, such as “urban area”, “region”, and “national” were included in the stop word list. This dictionary comprises 162 Chinese stop words, which can be utilized for preprocessing the Chinese corpus. These non-contributory words were removed from the text analysis by referencing the stop word list.

### Choosing the number of topics

**Fig 3:**
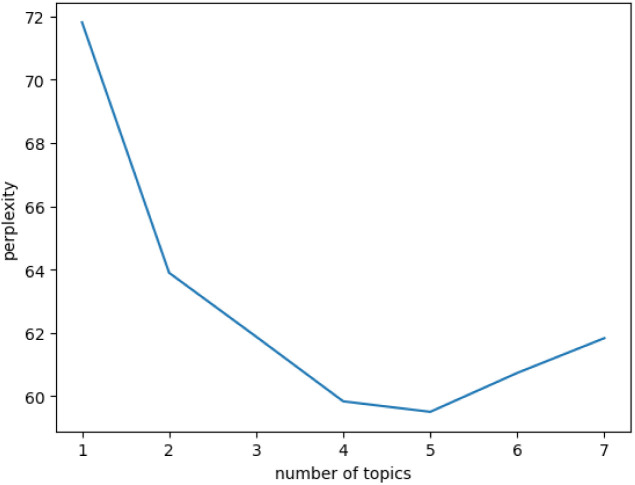
Perplexity score for different number of topics.

The selection of the optimal number of topics in topic model is crucial [27]. Perplexity is one of the most commonly used methods for evaluating topic models [28], and it has been employed to determine the optimal number of topics [24]. A lower perplexity score indicates better generalization performance of the model [24]. This study utilizes the perplexity function to measure the perplexity score using Python, and the resulting score is presented in Fig 3.

As shown in Fig 3, the perplexity exhibited the most significant decrease at around five topics, followed by incremental increases. Consequently, this study identifies five as the optimal number of topics.

### Topic extraction and labeling

**Fig 4:**
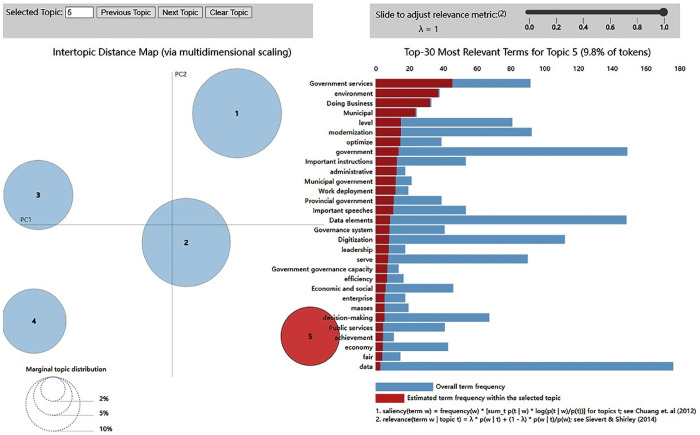
LDA model visualization.

This study utilized Python’s LDA visualization to extract topics concerning the factors influencing the adoption of the CDO. The identified topics and their associated keywords are summarized in Fig 4. As illustrated in Fig 4, the five topics are displayed alongside their top 30 words and corresponding relative weights.

This study assigns names to each topic based on the logical connection between its top words. The five topics, along with their most frequently occurring words, are presented in Table 1. As illustrated in Table 1, the five identified topics include data exploitation, data sharing, data management, data dividends and institutional pressures.

**Table 1:** Topics and the top 15 probable words for each topic.

Topics	Top 15 probable words
Data exploitation	chief data officer, institution, mechanism, data elements, data, exploitation modes, public data, public services, elements, digital economy, smart city, decision, system, high quality, exploitation system
Data management	government affairs, services, data, management, government services, standard, technology, digit, government data, decision, government, big data, business, institution, resources
Data sharing	digit, government, digitization, modernization, a coordination mechanism for government data sharing, strategy, digital China, national economy, digital economy, ecology, governance system, idea, people, rule of law, resources
Institutional pressures	government services, environment, doing business, municipal Party committee, standard, modernization, optimization, government, important instructions, administration, municipal government, work deployment, provincial government, important speech, data elements
Data dividend	data elements, data, digital ecology, market, industry, mechanism, elements, institution, public data, data security, ecology, high quality, digit, value, resources

### Research models

**Fig 5:**
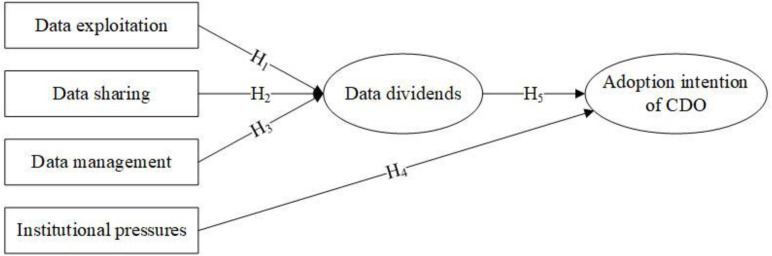
Research model.

Based on the five topics extracted from the topic model, the research model depicted in Fig 5 was developed. This conceptual model outlines the five factors hypothesized to influence the adoption of the CDO in government. Data exploitation, data sharing, and data management are identified as key antecedents for realizing data dividends. Furthermore, institutional pressures and data dividends are posited to significantly affect the adoption of the CDO.

### Data exploitation

Data exploitation can be understood as an organization’s utilization of analytical methods and techniques to generate valuable insights from data [29]. The key factor in reaping the benefits of big data lies in the effective and efficient utilization of data [30]. Data exploitation represents a crucial stage in the process of adding value to data [31]. An organization’s capability to exploit data directly influences the value it generates [32,33]. Previous research has confirmed that leveraging big data can promote both process and product innovation [34]. In various scenarios, governments develop new services for the public based on data exploitation, thereby enhancing the public good [33]. This leads to the formulation of the first hypothesis:

*H1. Data exploitation positively impact on data dividends.*

### Data sharing

Data sharing is defined as the “transfer of data between two or more organizations or individuals” [35]. With the advent of the internet, the topic of data sharing has gained prominence [36] and has become an increasingly an important area of research in public administration [37]. It serves as a crucial driver of transformational government [38] and is a defining characteristic of contemporary organizations [36]. Furthermore, data sharing constitutes a fundamental component of infrastructures [39]. The benefits of data sharing extend to both government organizations and external stakeholders [40]. For governments, these benefits include increased efficiencies, improved services, enhanced coordination among government organizations, and progressive social interventions [38,41]. In terms of stakeholders, the benefits comprise reduced data requests and opportunities for co-production with the government, among others. [37,42]. However, the absence of data sharing can lead to negative consequences [35]. Based on the insights provided in these studies, the following hypothesis is established:

*H2. Data sharing positively impact on the data dividends*

### Data management

Data management refers to the business function of planning, controlling, and presenting data and information [43,44]. It is a key research topic within the field of information systems [45]. Furthermore, data management is an important component of data resource governance [46] and the evaluation of e-government [47]. It has emerged as a means to assess the efficiency of existing processes and methods, and make changes accordingly [1]. Effective data management processes empower government to capitalize on data-driven opportunities [48]. The benefits of data management for government include improved efficiency [2,49], reduced decision- making costs [2], established data ownership [50], enhanced data analysis [1,51], increased data quality [46,52] and optimized data exchange [1]. Hence, the following hypothesis is proposed:

*H3. Data management will have a positive impact on the data dividends.*

### Institutional pressures

Institutional pressures are an important component of institutional theory [53]. Institutional pressures are defined as the pressures from the institutional environment that encourage organizations to adopt shared norms and routines [54]. Public organizations are particularly vulnerable to these pressures due to environmental uncertainty and challenges in assessing outputs [55]. Empirical literature has demonstrated that institutional pressures positively influence the adoption of e-government [55,56], open data [53], international public sector accounting standards [57], and accrual accounting [58]. Governments are likely to comply with institutional pressures [59], even in the absence of substantial empirical evidence suggesting that the CDO will improve government efficiency. The trend of appointing CDOs has generated institutional pressures that align with existing government data practices. Additionally, centralized countries experience stronger institutional pressures [60]. Therefore, the following formal hypothesis is formulated:

*H4. Institutional pressures will have a positive effect on the adoption of the CDO.*

### Data dividends

The concept of data dividends remains a topic of debate among researchers [61]. According to previous studies [61–63], the data dividend refers to the value derived from data use. The extent to which these values are realized is contingent upon the adoption of the CDO. To enhance the realization of data value, the CDO was introduced within government [3–5]. The primary responsibility of the CDO is to develop and implement data-related policies and to manage data effectively [5]. CDOs facilitate data sharing and flows, thereby enabling the realization of a data dividend. Consequently, the following hypothesis is formulated:

*H5. Data dividends will have a positive effect on the adoption of the CDO.*

## Research method

To test the hypotheses and provide data for the conceptual model, a survey method was employed for data collection. Questionnaires were distributed to employees in government organizations. SEM was utilized to evaluate the hypotheses outlined in the conceptual model.

### Construct operationalization

All measures for constructs were developed through successive stages of literature review, incorporating insights from both researchers and practitioners, followed by a process of refinement. To facilitate cumulative knowledge, the operationalization of constructs from previous research was used. Given that this study focuses on the factors influencing the adoption of CDO in government, some modifications were made to the existing scale. A five-point Likert scale (1 = strongly disagree, 5 = strongly agree) was employed for all items.

Data exploitation was measured using three-item scales adapted from Lee et al. [64]. Data sharing was measured with four-item scales derived from Li et al. [65]. Data management was measured through three-item scales drawn from Brinch et al. [66]. Institutional pressures were measured using four-item scales sourced from Ye et al. [67]. Adoption intention was measured using four-item scales based on Pappas et al. [68]. Data dividends was measured using four-item scales specifically designed for this study, informed by Lee et al. [69] and Lee et al. [5]. All scales were rephrased to specifically address the context of the CDO in government.

### Data collection

Data were collected from the sample through a questionnaire survey. To investigate the factors in the model, a questionnaire was developed based on prior relevant studies. This questionnaire includes demographic characteristics and measurement scales for the research variables. Additionally, the questionnaire was translated into Chinese.

This study did not require further ethics committee approval, since it neither involved animal or human clinical trials nor breached ethical standards. In accordance with the ethical principles outlined in the Declaration of Helsinki, all participants granted informed consent prior to engaging in the study. The anonymity and confidentiality of the participants were ensured, and participation was entirely voluntary.

The survey was conducted among MPA students and their colleagues. Initially, the hyperlink to the online survey was distributed in MPA WeChat groups, inviting MPA students to participate. Subsequently, these students forwarded the link to their colleagues, encouraging them to complete the questionnaire. A total of 295 questionnaires were returned, of which 18 were deemed invalid due to incompleteness. Therefore, the number of usable questionnaires was 277. The demographic data of the respondents is presented in Table 2.

**Table 2:** Demographic data of respondents.

Measure	Items	Frequency	Percentage
Gender	Female	135	48.7
	Male	142	51.3
Age	21–30	154	55.6
	31–40	82	29.6
	41–50	30	10.8
	51–60	11	4
Education	High school level	21	7.6
	University level	181	65.3
	Post-graduate	75	27.1
Work experience	1–5 years	86	31.05
	6–10 years	64	23.1
	11years and above	127	45.85

As shown in Table 2, the gender distribution among respondents was 51.3% male and 48.7% female. More than 50% of the respondents were aged between 21 and 30 years, while 29.6% were aged 31–40 years. The educational background of the respondents is as followings: 7.6% had completed high school, 65.3% held a university degree and 27.1% had obtained a postgraduate qualification. Regarding work experience, 31.01% of respondents had 1–5 years of experience, 23.1% had 6–10 years, and 45.85% had 11 years or more.

## Data analysis and results

### Scale validation

Validating a scale involves testing its reliability, as well as convergent and discriminant validity [70]. Construct reliability and convergent validity were commonly evaluated using Cronbach’s alpha values and factor loadings. The results, presented in Table 3, indicate that six constructs in the model showed good reliability with alphas values exceeding 0.8. Furthermore, all factor loading values ranged from 0.636 to 0.896 and were statistically significant at p = 0.001. Therefore, the criteria for construct reliability and convergent validity were satisfied.

**Table 3:** Measure scales and convergent validity.

Construct	Measure	Factor Loadings	CR	AVE
Data exploitation (DE)	If with the help of CDO, our organization can reuse existing data resources.	0.896***	0.877	0.705
	If with the help of CDO, our organization can reuse existing data applications and services.	0.820***		
	If with the help of CDO, our organization can reuse existing data skills.	0.799***		
Data sharing (DS)	Our organization and partners (e.g., other government organizations, business, the public) exchange relevant data with the help of the CDO.	0.812***	0.876	0.639
	Our organization and partners (e.g., other government organizations, business, the public) exchange timely data with the help of the CDO.	0.813***		
	Our organization and partners (e.g., other government organizations, business, the public) exchange accurate data with the help of the CDO.	0.758***		
	Our organization and partners (e.g., other government organizations, business, the public) exchange complete data with the help of the CDO.	0.814***		
Data management (DM)	CDO can help our organization advanced algorithms, tools and applications for data analysis.	0.874***	0.890	0.730
	CDO can help our organization establish and maintain an integrated data- and system network.	0.837***		
	CDO can help our organization create governance procedures of harmonizing and cleaning of data.	0.851***		
Institutional pressure (IP)	The superior government requires our organization to create the CDO.	0.835***	0.822	0.538
	Other governments earlier implementation of CDO provides a benchmark and guidance for our organization’s CDO.	0.636***		
	The intense competition between governments exerts strong pressures on the creation of CDO in our organization.	0.740***		
	The public and business have a strong influence on the creation of CDO in our organization.	0.709***		
Data dividends (DD)	CDO can improve government efficiency	0.753***	0.880	0.647
	CDO can improve public service quality	0.806***		
	CDO can form data driven decision-making	0.855***		
	CDO can improve data exchange	0.800***		
Adoption intention (AI)	Our organization is planning to adopt CDO	0.842***	0.880	0.648
	Our organization is willing to adopt CDO	0.761***		
	I think that our organization will adopt CDO	0.793***		
	I predict that our organization should adopt CDO	0.822***		

*** p < 0.001.

Discriminant validity was assessed based on the criteria proposed by Fornell and Larcker [70]: the square root of the average variance extracted (AVE) must be greater than the correlations between the construct and other constructs within the model. The results presented in Table 4 demonstrate the AVEs for all constructs surpassed their corresponding cross-correlations, thereby confirming that the criteria for discriminant validity have been met.

**Table 4:** AVE and correlation of latent variables.

Construct	AVE	Factor correlation					
		DE	DS	DM	IP	DD	AI
DE	0.705	**0.840**					
DS	0.639	0.737	**0.799**				
DM	0.730	0.773	0.754	**0.854**			
IP	0.538	0.384	0.377	0.351	**0.733**		
DD	0.647	0.645	0.647	0.667	0.454	**0.804**	
AI	0.648	0.555	0.532	0.570	0.490	0.591	**0.805**

### Model testing results

The structure model was tested using SEM conducted in AMOS 25. As summarized in Table 5, χ^2^/df (χ^2^ = 242.905, df = 198) was 1.227, which is less than the threshold of 3.0. Furthermore, NFI, GFI and CFI all exceeded 0.9, while RMSEA was 0.029, which is below the acceptable limit of 0.1. These results indicate that the model-fit surpassed the commonly accepted standards, suggesting that all the indices demonstrate a very good fit.

**Table 5:** Overall model-fit indices for the research model.

Model-fit indices	Results	Recommended value
Chi-square statistic χ2/df	1.227 (242.905/198)	≤3
NFI	0.938	≥ 0.9
GFI	0.929	≥ 0.9
CFI	0.988	≥ 0.9
RMSEA	0.029	< 0.1

### Hypothesis testing

**Fig 6:**
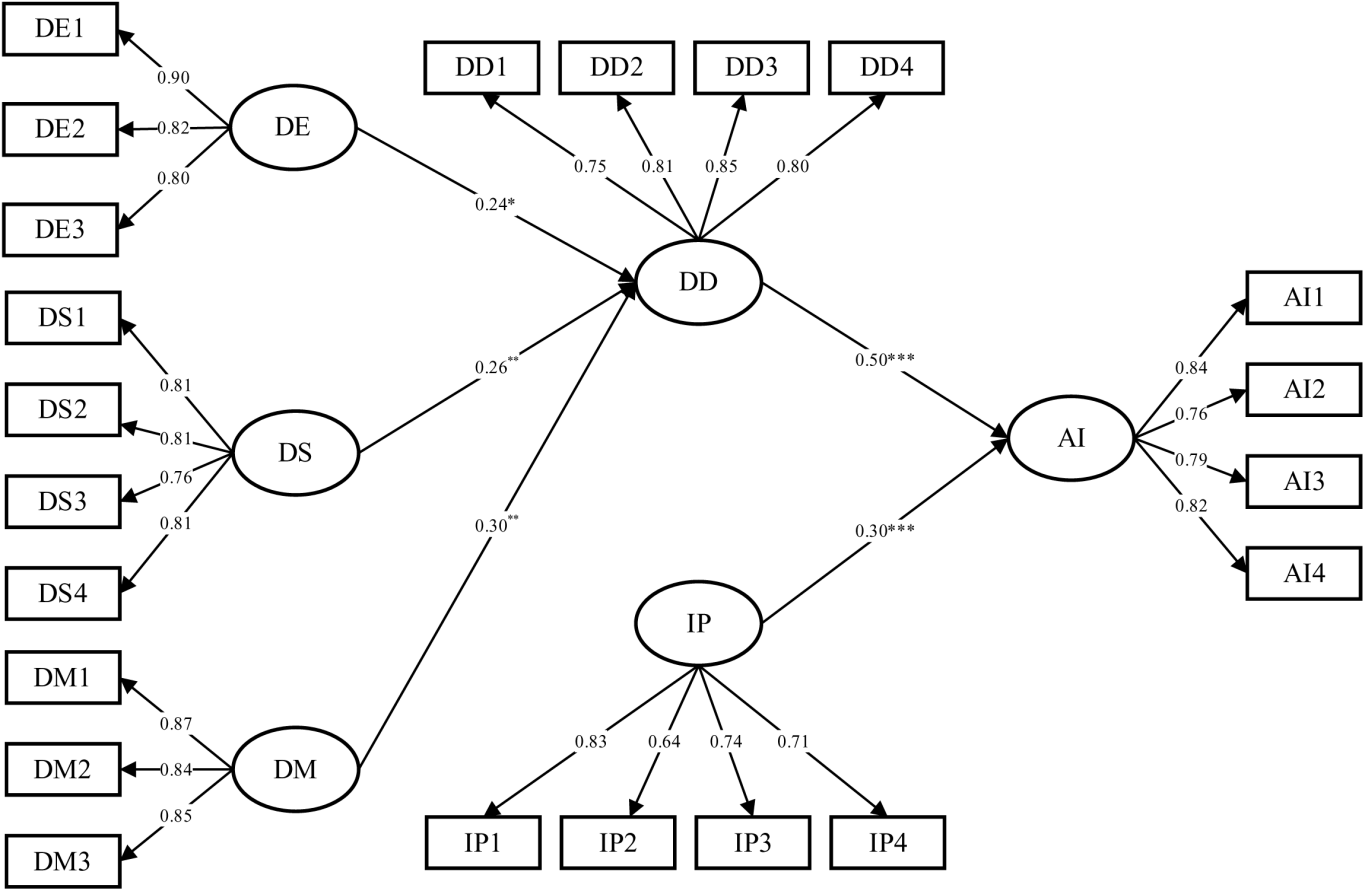
SEM analysis of the research model.

The hypotheses were collectively tested by examining the significance of the relationships in the SEM model. The path significance of each hypothesized association in the research model along with the explained variance (R^2^ value) were examined. Fig 6 shows the standardized path coefficients and their corresponding significance levels for the research model.

All paths illustrated in Fig 6 were found to be significant. Specifically, data exploitation (β = 0.239, ρ = 0.017 < 0.05), data sharing (β = 0.257, ρ = 0.008 < 0.01) and data management (β = 0.305, ρ = 0.003 < 0.01) each demonstrated a significant effect on data dividends. Moreover, data dividends (β = 0.498, ρ = 0.000 < 0.001) and institutional pressures (β = 0.299, ρ = 0.000 < 0.001) significantly influenced the adoption intention of the CDO. As shown in Table 6, five hypotheses in the research model were supported.

**Table 6:** Research hypothesis testing results.

Research Hypothesis	T-Value	ρ	β	R^2^	Support or not
DE → DD	2.406	*	0.239	0.536	Support
DS → DD	2.660	**	0.257		Support
DM → DD	2.941	**	0.304		Support
DD → AI	7.508	***	0.498	0.429	Support
IP → AI	4.813	***	0.299		Support

*** p < 0.001; ** p < 0.01; * p < 0.05.

## Discussion

Existing research on CDO has primarily focused on general descriptions of CDO. However, little research has explored the reasons behind the establishment of the CDO. This study employs topic model to identify the factors influencing CDO adoption and SEM to validate the research model. The findings suggest that data exploitation, data sharing and data management significantly influence data dividends. Moreover, data dividends and institutional pressures have a significant impact on the intention to adopt CDO, with digital dividends exerting a greater effect than institutional pressures.

The results of the study indicate that data exploitation is a significant antecedent of data dividends in government. This finding aligns with and empirically supports the conclusions of previous studies [32,33]. Data exploitation serves as a pathway for the CDO to unlock the value of data. It can lead to the development of new data products and services that meet the needs of both the public and businesses. The purchase of these data products and services by the public and businesses not only realizes the value of the data but also motivates government departments to provide more high-quality data for exploitation. Since their inception, CDOs have provided tools and models for data exploitation through the construction of various digital systems and platforms. Additionally, data quality issues have posed a significant obstacle to data exploitation, and CDOs enhance data quality through effective data governance.

The study also shows that data sharing has a significant positive impact on data dividends. This finding is consistent with and empirically supports the results of previous studies [37,38,41,42,71]. Data sharing is influenced by numerous factors, among which organizational factors play a crucial role [35,37]. It not only serves as a catalyst for the creation of CDO, but also enables CDOs to recognize the value of data. Building upon the initial government data openness and sharing, CDOs can further enhance and promote the openness and sharing of government data resources. The practices of CDOs can improve coordination mechanisms and foster innovative models for openness and sharing of government data resources. Consequently, governments, the public, and enterprises can obtain necessary information by analyzing government data resources.

The study also found that data management is a predictor of data dividends. This result aligns with and empirically support the findings of previous studies [1,2,49]. The government is transitioning from a traditional model to a data-driven approach, with CDO acting as enablers in this transformation process. Concurrently, CDOs have been improving leadership and the capacity to effectively manage government data resources. Emerging mechanisms for data-driven management, innovation and decision-making are becoming increasingly apparent. Additionally, an efficient organizational management system for the management of government data resource is being developed. Collectively, these developments will enhance data management and data quality, ultimately leading to a more effective exploitation of data value.

It is also important to consider the positive relationship between institutional pressures and the intention to adopt the CDO. This finding is consistent with the results of previous studies [53,55,56]. Institutional pressures can be categorized into coercive, mimetic and normative pressure [72,73]. As data increasingly becomes a strategic asset for governments [2], the central government has enacted policies and regulations concerning digital government, big data development, public data, and data elements. These policies and regulations specifically emphasize the role of the CDO or reference it directly. Moreover, some provincial governments have formalized policies regarding the CDO. In 2021, Guangdong province pioneered the introduction of the first provincial-level CDO policy, known as the Pilot Program for the CDO System in Guangdong Province. In centralized countries, there tends to be stronger coercive pressure [60], which encourages municipal governments to adopt the CDO. Additionally, competition among governments can create mimetic pressure for those that have not yet adopted the CDO. Due to issues related to data quality and insufficient data supply, meeting the needs of the public and enterprises becomes challenging, thereby creating normative pressure on governments that do not adopt the CDO.

The study further demonstrates that data dividends significantly positively influence the intention to adopt the CDO. Data dividends serve as a crucial driver for the establishment of the CDO. The development of digital government and cultivation of a high-quality economy are facilitated by the release of data element dividends. Consequently, data dividends incentivize the government to adopt the CDO.

### Implications

#### Implications for theory.

First, this study makes a significant contribution to the literature regarding the adoption of the CDO in government. Several authors have called for further research on the CDO [13,74], highlighting a gap in the existing literature. Thus, this study aims to investigate the underlying reasons behind the adoption of the CDO in government. Using factors identified through topic model, this study conducts a quantitative analysis of five factors influencing the intention to adopt the CDO. The findings yield a parsimonious model that elucidates the reasons for the adoption of the CDO in government.

Second, the concept of data dividends is described in a limited body of literature. Furthermore, there is currently no established measurement scale for data dividends. Thus, drawing on previous studies and the specific research context, this study develops a four-item scale to measure data dividends. This measurement scale is intended for use in research related to CDO in the governmental context.

#### Implications for practice.

The findings of this research have significant practical implications for government organizations aiming to enhance the adoption of the CDO. First, an understanding of the key factors influencing the adoption of the CDO will enable practitioners to formulate effective policies that encourage government organizations without a CDO to embrace this position. Such policies can provide essential support, guidance, and incentives to facilitate effective government operations and employee training. For instance, the government could establish benchmark departments, allowing those without a CDO to visit and learn from the experiences and practices of these established departments.

Second, data dividends have emerged as a crucial variable influencing the effective adoption of the CDO in government. The realization of data dividends depends on various data related work, such as data exploitation, data sharing and data management. Superior government organizations implement appropriate strategies and well-designed training systems to enhance awareness among departmental leaders. This approach helps them recognize the significance of data management and its profound impact on both society and the economy, thereby enhancing their enthusiasm to establish CDO. Furthermore, government organizations must establish standards and rules related to data management, which will enable staff to operate in accordance with these standards.

## Limitations and further research

This study has several limitations. First, while it examined five factors as antecedents to the adoption the CDO in government, there are additional factors that may also influence this adoption. Factors such as government readiness, human resources, and leader’s awareness could significantly impact the adoption of the CDO. Future research should consider extending the model to incorporate these and other relevant variables. Second, the large sample size of the current study enhances the generalizability of its findings. Moreover, future research should expand the investigation to include government organizations in other countries to further enhance the generalizability of the results. Finally, due to the limitations of SEM, this study primarily focused on the linear relationships among variables. However, the actual relationships between the five influencing factors and the adoption intention of the CDO may be more complex, potentially involving non-linear relationships [75]. These non-linear relationships were not thoroughly examined in this study, suggesting that future research should take into account the effects of such non-linear more thoroughly.
